# Luminal epithelium remodeling underlies endometrial regeneration during menstruation and pregnancy

**DOI:** 10.64898/2026.03.08.710375

**Published:** 2026-03-10

**Authors:** Claire J. Ang, Jaina L. R. Gable, Kayla C Lyons, Enya Miguel Whelan, Çağrı Çevrim, Taylor D. Skokan, Sofia G. Bennetts, Benjamin D. Manetta, Aellah M. Kaage, Dhanvika Mopure, Ana Breznik, Patricia L. Murphy, Allison E. Goldstein, Fátima Sanchís-Calleja, Thomas E. Spencer, Andrew M. Kelleher, Kara L. McKinley

**Affiliations:** 1 Department of Stem Cell and Regenerative Biology, Harvard University, Cambridge MA, 02138, USA; 2 Department of Molecular and Cellular Biology, Harvard University, Cambridge MA, 02138, USA; 3 Howard Hughes Medical Institute, Cambridge MA, 02138, USA; 4 Biological and Biomedical Sciences Program, Harvard University, Cambridge MA, 02138, USA; 5 Department of Obstetrics, Gynecology, and Women’s Health, University of Missouri, Columbia MO, 65211, USA; 6 Division of Animal Sciences, University of Missouri, Columbia MO, 65211, USA.

## Abstract

Menstruation and pregnancy disrupt substantial proportions of the uterine lining (endometrium). These breaches impose an immense regenerative burden on the luminal epithelium that lines the uterine cavity, which is proposed to be replenished by cells residing in adjoining epithelial glands. Here, we show that the luminal epithelium and glandular epithelium are maintained by separate progenitor populations during homeostasis, induced menstruation, pregnancy, and postpartum repair in mice. These data challenge the gland-centric model of regeneration during these physiological events, although we find that gland cells can resurface the tissue after chemical ablation. Our data indicate that during menstruation, the luminal epithelium bypasses the need for gland contributions by undergoing extensive expansion and morphogenesis to re-epithelialize stromal surfaces as the tissue breaks down. Analogous morphogenesis occurs during gestational remodeling, revealing luminal epithelial expansion as a unifying mechanism enabling simultaneous stromal disruption and re-epithelialization, which may underlie the endometrium’s remarkable resilience to fibrosis.

## Graphical Abstract

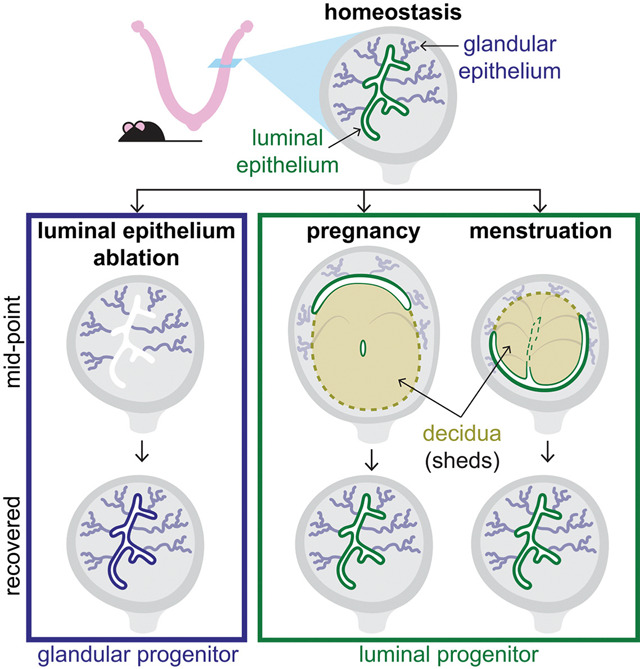

## Introduction

**Figure 1.**
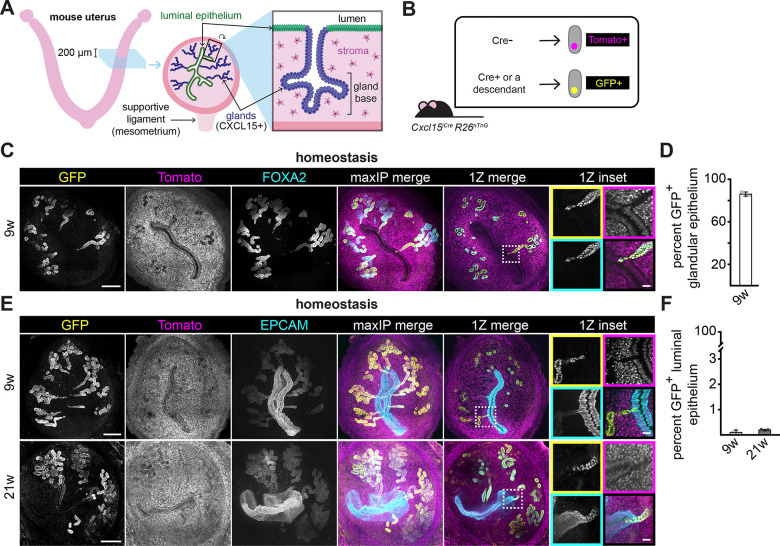
Endometrial gland lineages make minimal contributions to the luminal epithelium in homeostasis (A) Schematic of mouse uterus, and 200 *μ*m thick transverse cross-section. All sections in the manuscript are presented with the mesometrium down. (B) Schematic of the resulting TdTomato+ nuclear staining for Cre-naïve lineages and GFP-positive nuclear staining in Cre-exposed lineages from *Cxcl15*^*iCre*^ *R26*^*nTnG*^ mice. (C) Representative immunofluorescence images of *Cxcl15*^*iCre*^ *R26*^*nTnG*^ uteri collected at 9 w of age. (D) Quantification of the proportion of FOXA2+ (gland) nuclei expressing GFP in *Cxcl15*^*iCre*^ *R26*^*nTnG*^ uteri collected at 9 w of age. n = 3. (E) Representative immunofluorescence images of *Cxcl15*^*iCre*^ *R26*^*nTnG*^ uteri collected at 9 or 21 w of age. LUTs were adjusted independently for each condition to maximize signal-to-noise. (F) Quantification of the proportion of luminal epithelium nuclei positive for GFP in *Cxcl15*^*iCre*^ *R26*^*nTnG*^ uteri collected at 9 or 21 w of age. n = 3 per condition. For all images, single channel outsets are shown are as maxIPs. Inset channel is indicated by box color. For all graphs, each dot represents an individual animal and error bars represent standard deviation. Outset scale bars, 200 *μ*m; inset scale bars, 50 *μ*m.

The endometrial tissue that lines the uterine cavity undergoes dramatic remodeling and disruption during menstruation, pregnancy and childbirth (parturition). During the human menstrual cycle, hormone changes stimulate the endometrium to differentiate into a specialized tissue called the decidua. If pregnancy occurs, the decidua persists and remodels around the embedding embryo and its developing placenta until delivery. In the absence of pregnancy, the decidua is instead shed during menstruation. Each of these events perturb the endometrium’s major structural components, including a luminal epithelium, branching epithelial glands, and the surrounding supportive stroma (Figure 1A). Proper restoration of these components is crucial for fertility, as the luminal epithelium serves as a mediator of decidualization and the site of initial embryo attachment,^1,2^ and the glands secrete factors to support early pregnancy.^3–5^ Moreover, the restoration of endometrial epithelial populations is critical to guard underlying stromal fibroblasts against inflammatory signals associated with scar formation and loss of tissue function.^6^ However, our understanding of the progenitor dynamics and cellular mechanisms underlying rapid endometrial epithelial regeneration across these events remains incomplete.

The bases of the glands have been proposed as a key reservoir for endometrial epithelial progenitor cells in humans. This model is particularly appealing in the context of menstruation, which is thought to occur in a stepwise manner, in which shedding of the superficial endometrium (the functionalis) exposes an underlying endometrial layer (the basalis), comprised of gland fragments and remnant stroma. The basalis glands are enriched in putative stem cell markers and features associated with stemness, such as the capacity for clonogenicity and self-renewal *in vitro*.^7–14^ One study examining the human endometrium found that spontaneously arising mutant clones in the basalis glands expand to the functionalis glands with age, supporting a role for basalis progenitors in regenerating the upper glands.^15^ However, the contributions of basal gland progenitors to repopulating the luminal epithelium were not extensively assessed.

Due to the challenge of discerning the molecular identities of progenitors in situ in humans, several studies have performed lineage tracing in mice to dissect the roles of glandular epithelial cells during endometrial homeostasis and regeneration.^16–18^ However, these studies have been inconclusive. Lineage tracing gland populations using genetic drivers ubiquitously expressed in glands (*Lgr5* and *Foxa2*) revealed no contribution to the luminal epithelium during adulthood, even after 6 weeks or 26 weeks, respectively.^17,18^ In contrast, a specialized subset of glandular cells expressing *Axin2* was recently found to contribute to the luminal epithelium within 12 weeks of inducing labeling in adults.^16^ The effect of menstruation on progenitor dynamics was not assessed in these studies, as mice do not menstruate naturally. However, menstruation-like endometrial shedding can be induced in mice through a variety of methods combining experimental hormone control with the addition of a decidualization stimulus.^19^ Defining the contributions of glandular cells to the luminal epithelium across menstruation, pregnancy, and parturition is a critical open question that requires further investigation.

Here, we use lineage tracing in mice to track gland contributions to the luminal epithelium across multiple endometrium disruption events, including luminal epithelium ablation, menstruation, pregnancy and parturition. Our analyses revealed that gland cells made minimal contributions to the luminal epithelium during homeostasis but could assist in regeneration upon near total loss of luminal epithelium through chemical ablation. Gland contributions to the luminal epithelium were also minimal during induced menstruation, pregnancy and parturition. During induced menstruation, we found that persistent sheets of luminal epithelium undergo extensive growth, enveloping the decidua and re-epithelializing the lumen prior to shedding. This process bypasses the need for gland contributions and achieves endometrial repair concurrently with tissue detachment, in contrast to the assumed progression of menstruation through tissue denudation followed by resurfacing of the exposed stroma. We identified analogous luminal epithelium morphogenesis occurring during gestation, which enables the endometrium to appropriately compartmentalize the fetal tissues deeply embedded in the underlying stroma. These findings reveal luminal epithelial expansion concomitant with tissue disruption as a unifying mechanism by which the uterus achieves rapid epithelial restoration in menstruation and pregnancy.

## Results

### Glandular lineages only contribute to the luminal epithelium upon extensive chemical ablation

We first sought to test the contributions of gland progenitors to unperturbed luminal epithelium during tissue turnover. For these experiments, we employed the recently developed *Cxcl15*^*iCre*^ *R26*^*nTnG*^ mouse line, which induces a permanent, heritable switch from TdTomato to GFP expression in cells expressing *Cxcl15* (Figure 1B).^20^ *Cxcl15* expression is specifically detected in the glandular epithelium by postnatal day 12, when the glands first form by budding from the luminal epithelium. In contrast, expression is not detected in the luminal epithelium.^20^ In young adults (9 weeks old), 86% of cells expressing the glandular marker, *Foxa2*, were GFP-positive, indicating robust labeling of glandular epithelial cells (Figure 1C and 1D). In the endometrium of cycling mice, luminal epithelial cells proliferate every 3 days.^16^ Despite this high turnover, only 0.09% and 0.19% of luminal epithelial cells expressed GFP at 9 and 21 weeks of age, respectively (Figure 1E, 1F and Table S1). These data demonstrate that the glandular epithelium makes minimal contributions to the luminal epithelium during homeostasis.

**Figure 2.**
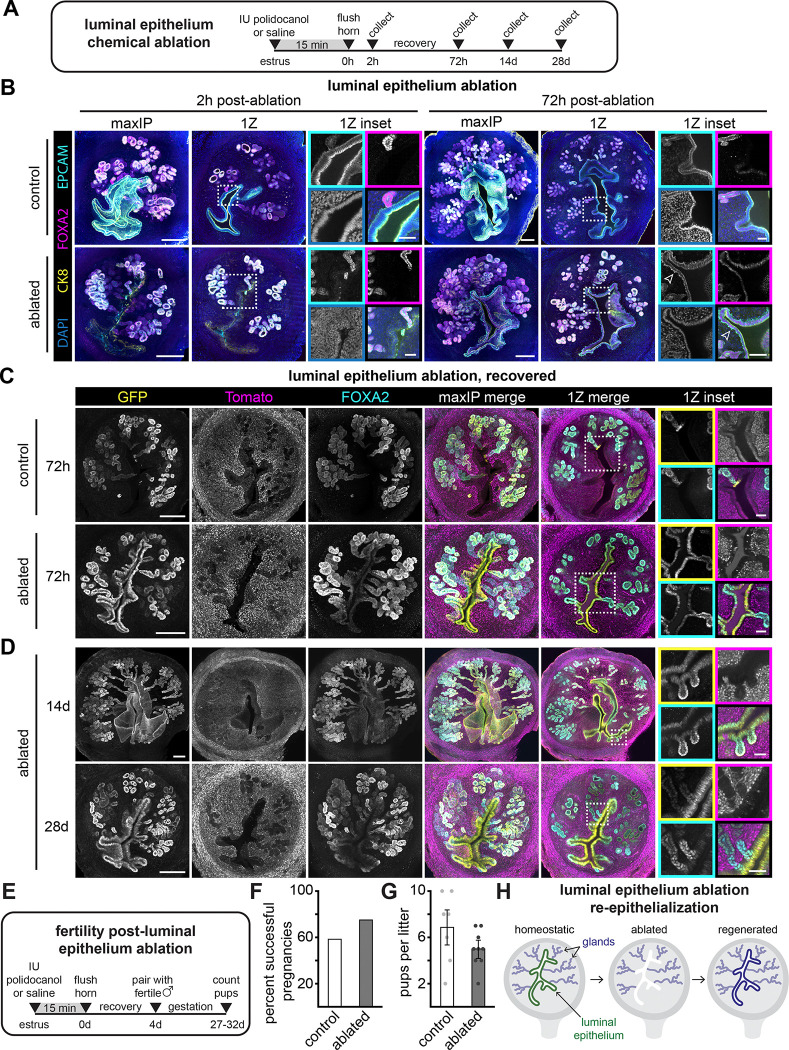
Luminal epithelium ablation stimulates gland lineage contributions (A) Experimental timeline for luminal epithelium chemical ablation in C57BL/6 or *Cxcl15*^*iCre*^ *R26*^*nTnG*^ mice. (B) Representative immunofluorescence images of C57BL/6 uteri collected as indicated in A. Arrowhead outlines indicate root-like membrane protrusions from the basal side of new luminal epithelium. (C) Representative immunofluorescence images of *Cxcl15*^*iCre*^ *R26*^*nTnG*^ uteri collected as indicated in A. Single Z plane and inset LUTs were adjusted independently for each condition to maximize signal-to-noise. (D) Representative immunofluorescence images of *Cxcl15*^*iCre*^ *R26*^*nTnG*^ uteri collected as indicated in A. (E) Experimental timeline for testing fertility in C57BL/6 mice following luminal epithelium ablation or saline injection. (F) Quantification of the proportion of C57BL/6 females that carried a litter to term following recovery from ablation or saline injection. n = 12 per condition. (G) Quantification of the number of pups per litter from C57BL/6 dams following recovery from ablation or saline injection. Each dot represents a litter and error bars represent standard deviation. n = 7 control; n = 9 ablated. (H) Summary schematic of recovery from luminal epithelium ablation. For all images, single channel outsets are shown are as maxIPs. Inset channel is indicated by box color. Outset scale bars, 200 *μ*m; inset scale bars, 50 *μ*m. CK8, cytokeratin 8; IU, intrauterine.

Although luminal and glandular compartments were largely maintained by separate progenitors during homeostasis, we next considered the possibility that tissue disruption might enable cross-compartment plasticity, as occurs in other epithelial systems, including the skin.^21,22^ To test this, we sought to challenge the endometrium with extensive loss of luminal epithelium. We developed a chemical ablation approach using transient exposure to the liquid surfactant, polidocanol, which is used as a model of epithelial damage in the airway (Figure 2A).^23^ 2 h after polidocanol treatment, nearly all of the luminal epithelium and gland necks directly abutting the uterine cavity were ablated, while underlying glandular epithelium remained intact (Figure 2B). Within 72 h of polidocanol washout, the luminal epithelium had regenerated along the entire horn. To assess whether the repaired luminal epithelium was gland-derived, we repeated ablation experiments in *Cxcl15*^*iCre*^ *R26*^*nTnG*^ mice. 72 h after ablation, the luminal epithelium was almost entirely GFP-positive (gland-derived) throughout the length of the horn in most mice (Figure 2C). In one animal, we observed a mix of GFP-positive and -negative luminal epithelial cells, suggesting incomplete luminal epithelium ablation. The majority of luminal epithelial cells remained GFP-positive 14 and 28 days after polidocanol ablation, suggesting long-term persistence (Figure 2D). Together, our data indicate that glandular cells can give rise to luminal epithelial cells after extensive luminal epithelial ablation (Figure 2H).

Upon recovery from polidocanol treatment, the repaired luminal epithelium exhibited short, root-like cell membrane projections into the stroma, but appeared otherwise normal (Figure 2B). Additionally, we observed ubiquitous, persistent expression of *Foxa2* in the luminal epithelium, albeit at lower levels than the glands (Figure 2C and 2D). *Foxa2* is canonically restricted to the glands during adulthood and serves critical functions during postnatal gland development and fertility.^24,25^ In light of these aberrations, we evaluated the function of gland-derived luminal epithelium by testing its competence to facilitate pregnancy (Figure 2E). 75% of mice that underwent luminal epithelium ablation were able to carry litters to term, compared to 58% of unablated controls, although polidocanol-treated animals produced slightly fewer pups per litter (Figure 2F and 2G). Thus, although glandular lineages make minimal contributions during homeostasis, they have the capacity to form functional, albeit altered, epithelium upon extensive chemical ablation of the luminal epithelium.

### Persistent luminal epithelium covers shedding and retained tissue during induced menstruation

Our data indicated that two different progenitors can replenish the luminal epithelium in a context-dependent manner. In homeostasis, luminal populations were self-renewing, but after chemical ablation, glandular progenitors contributed to luminal epithelium regeneration. Thus, we sought to determine which of these progenitor populations contribute in response to physiologically relevant disruptions to the endometrium, beginning with menstruation.

**Figure 3.**
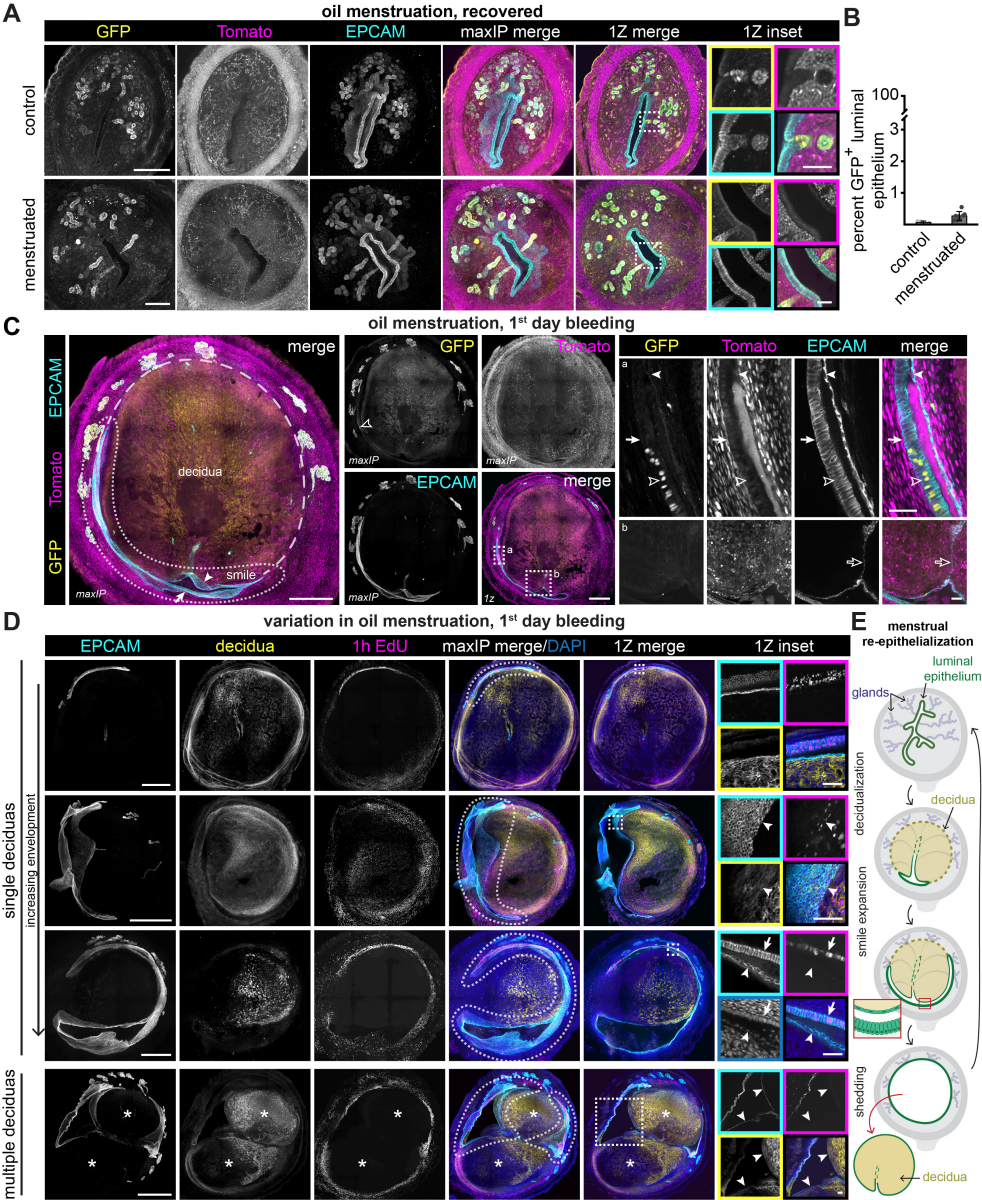
Luminal epithelium envelops decidualized tissue during induced menstruation (A) Representative immunofluorescence images of *Cxcl15*^*iCre*^ *R26*^*nTnG*^ uteri collected after recovery from oil-induced menstruation. See Figure S1A for the experimental timeline. LUTs were adjusted independently for each condition to maximize signal-to-noise. Scale bars represent 200 *μ*m. (B) Quantification of the proportion of luminal epithelium nuclei positive for GFP in *Cxcl15*^*iCre*^ *R26*^*nTnG*^ uteri after recovery from oil menstruation or saline injection. Each dot represents one animal and error bars represent standard deviation. n = 4 control; n = 8 menstruated. (C) Representative immunofluorescence images of *Cxcl15*^*iCre*^ *R26*^*nTnG*^ uteri collected on the first day of oil menstruation bleeding. (D) Immunofluorescence images demonstrating variation between uteri collected on the 1^st^ day of oil menstruation bleeding. P-cadherin (decidua marker) is shown in yellow. LUTs were adjusted independently for each sample. (E) Summary schematic of progression through decidualization, menstruation and recovery from oil-induced menstruation. For all images, single channel outsets are shown as maxIPs, and insets are shown as single Z planes. Inset channel is indicated by box color unless directly noted. Dotted lines indicate smile structures, dashed lines outline decidua, solid arrows indicate basal-side columnar luminal epithelium, solid arrowheads indicate squamous decidual-side luminal epithelium, outlined arrowheads indicate GFP-positive luminal epithelial cells, outlined arrows indicate a connection to the deteriorating lumen, and asterisks indicate individual decidua. Outset scale bars, 500 *μ*m, with the exception of panel A; inset scale bars, 50 *μ*m.

Menstruation is proposed to proceed through shedding of much of the luminal epithelium and upper stromal tissues, leaving behind an exposed stromal surface dotted with beheaded gland bases, akin to our chemical ablation model. Thus, we hypothesized that gland progenitors would regenerate the luminal epithelium after induced menstruation. We induced menstrual-like shedding in *Cxcl15*^*iCre*^ *R26*^*nTnG*^ mice by administering estrogen and progesterone to ovariectomized mice, then injecting oil into the uterine lumen to trigger decidua formation (Figure S1A). Although an experimentally induced decidua is sometimes referred to as a deciduoma, we use decidua to refer to both natural and artificially induced decidual masses throughout the text for simplicity. As the decidua requires continued progesterone to be maintained, the cessation of hormone injections in this model triggers decidual shedding, which is detectable as blood in vaginal lavages 2 to 4.5 days on average after progesterone withdrawal. Surprisingly, only 0.27% of luminal epithelial cells were GFP-positive after recovery from induced menstruation (10 days after the last progesterone injection; Figure 3A, 3B and Table S1). Therefore, we find that the glands are not major participants in luminal epithelium regeneration upon menstrual-like shedding.

Our data indicated that GFP-negative luminal epithelial progenitors may persist through menstruation rather than shedding entirely. To further interrogate the cell types present during menstrual shedding, we collected *Cxcl15*^*iCre*^ *R26*^*nTnG*^ uteri from mice undergoing oil-induced menstruation on the first day they exhibited blood in a vaginal lavage. At this timepoint, the decidua had pushed the gland bases to the periphery of the tissue, similar to previous reports of decidualization.^26,27^ We detected sheets of persistent, largely GFP-negative luminal epithelia, which contained a few rare patches of GFP-positive luminal epithelium (Figure 3C and S1B). These data illustrate that substantial luminal epithelial cell reservoirs persist during menstruation.

We observed that the GFP-negative luminal epithelium curved around the periphery of the decidualized tissue in 70% of oil-induced menstruation samples (76/108 sections, n = 3 mice). Based on their arched morphology, we dubbed these structures “smiles.” Smiles emerged along the future division plane of the tissue, where the decidua would soon detach. These structures consisted of two halves that exhibited distinct epithelial morphologies: a flattened (squamous) epithelium lined the surface of the deteriorating deciduoma (the “upper lip”) and a columnar epithelium lined the basal endometrium that persists during shedding (the “lower lip”, Figure 3C). Co-staining with the decidual marker P-cadherin^28^ revealed that smiles enveloped deciduas to different degrees within and between uteri (Figure 3D and S1B). As oil-induced menstruation can generate multiple decidual masses along the horn, some regions exhibited epithelium in the middle of the tissue, where two deciduas met (Figure 3D). A 1 h EdU pulse revealed extensive luminal epithelial proliferation at this time point (Figure 3D), suggesting a potential mechanism by which proliferation widens the smile over time. We detected equivalent smile structures in an alternative model of menstruation (X-Mens),^28^ which triggers decidua formation by chemogenetically activating the decidualization pathway (Figure S1C and S1D). Together, our data are inconsistent with a model of stepwise decidual tissue detachment and subsequent re-epithelialization. Rather, they indicate active luminal epithelial expansion and stromal resurfacing prior to loss of the decidua (Figure 3E).

### Luminal epithelium envelops decidualized tissue during gestational remodeling and resurfaces the endometrium post-partum

Like menstruation, pregnancy and parturition require extensive reorganization of the endometrial epithelium, but many details of these processes, including their progenitor dynamics, have not yet been elucidated. Recent work has revealed key features of epithelial remodeling during embryo implantation.^26,27,29^ However, later gestation also reshapes the epithelium dramatically, and these events have remained largely unexplored since an insightful study detailed the events of rat gestation over 40 years ago.^30^

**Figure 4.**
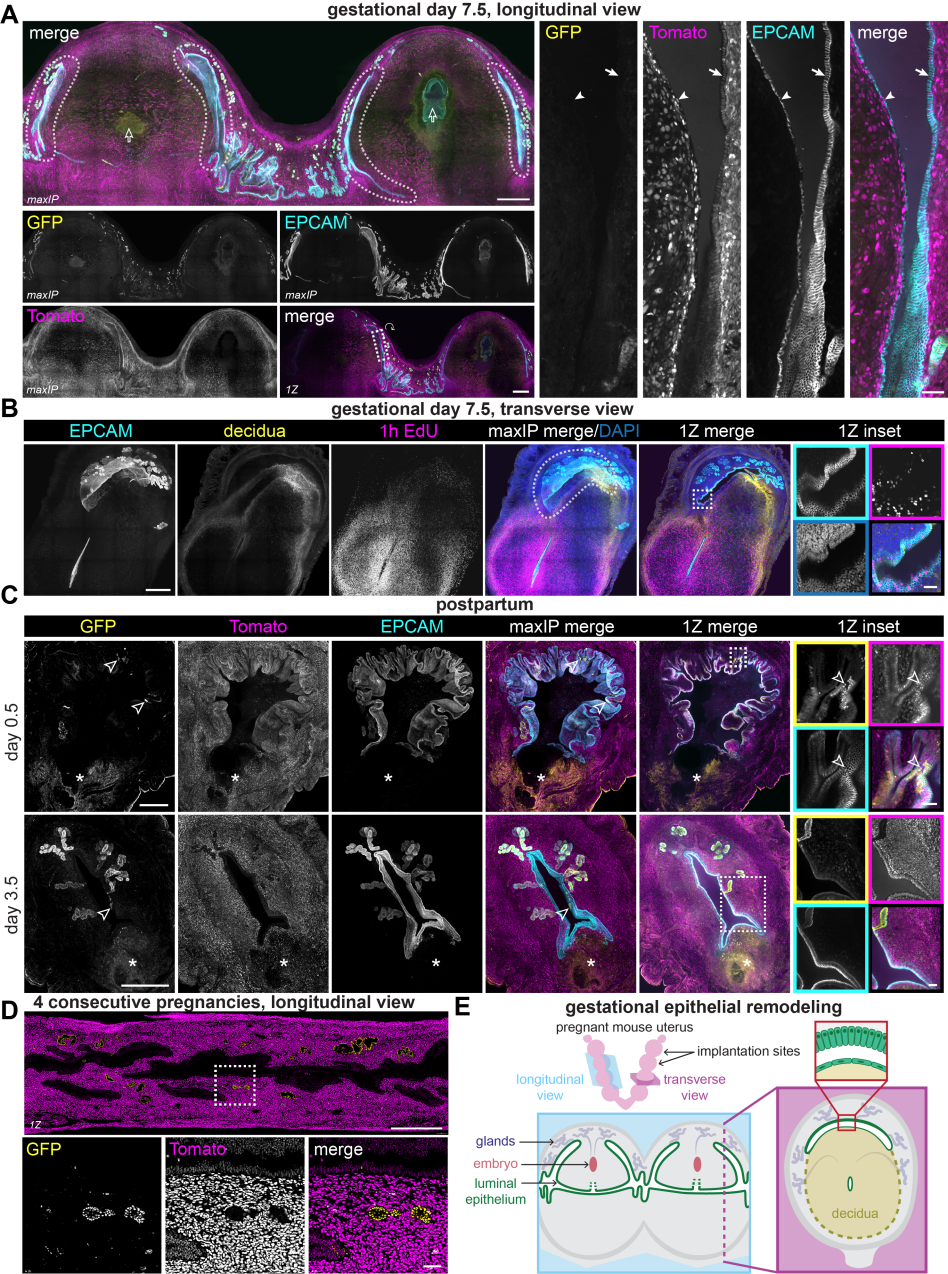
Luminal epithelium envelops decidualized tissue during gestation and restores epithelial integrity during postpartum repair (A) Representative immunofluorescence images of two adjacent longitudinally sectioned implantation sites in *Cxcl15*^*iCre*^ *R26*^*nTnG*^ uteri. See E for a representation of section orientations. (B) Representative immunofluorescence images of a transverse sectioned implantation site collected on gestational day 7.5. P-cadherin (decidua marker) is shown in yellow. (C) Representative immunofluorescence images of *Cxcl15*^*iCre*^ *R26*^*nTnG*^ uteri on postpartum day 0.5 or 3.5. LUTs were adjusted independently for each condition to maximize signal-to-noise. (D) Representative immunofluorescence images of a longitudinally cryosectioned *Cxcl15*^*iCre*^ *R26*^*nTnG*^ dam uterus collected after recovery from 4 consecutive pregnancies. (E) Summary schematic of luminal epithelium remodeling around implantation sites on gestational day 7.5, represented as longitudinal and transverse views. Single channel outsets are shown as maxIPs, and insets are shown as single Z planes. Inset channel is indicated by box color unless directly noted. Outlined arrows indicate embryos, solid arrows indicate basal columnar epithelium, solid arrowheads indicate squamous decidual-side epithelium, outlined arrowheads indicate GFP-positive luminal epithelial cells, and asterisks indicate placental detachment sites. Outset scale bars, 500 *μ*m; inset scale bars, 50 *μ*m.

To test for gland lineage contributions to the luminal epithelium by mid-gestation, we evaluated *Cxcl15*^*iCre*^ *R26*^*nTnG*^ dam uteri collected on gestational day 7.5. As in our induced menstruation samples, our analyses revealed extensive GFP-negative, proliferative epithelial smiles surrounding each discrete implantation site, with squamous epithelium lining the decidua, and columnar epithelium lining the basal endometrium (Figure 4A and 4B). We confirmed that these epithelial structures were of maternal rather than fetal origin by lineage tracing fetal tissues (Figure S2A and S2B). Continued expansion of these smiles later in gestation may underlie our observation that the tissue is almost completely resurfaced by luminal epithelial cells (GFP-negative) by the day of parturition, leaving behind only a small placental detachment site (Figure 4C). Indeed, a fortuitous natural case of fetal resorption collected at term revealed that the resorption site was almost completely enveloped in GFP-negative luminal epithelium (Figure S2C). We also observed minimal gland contribution to resurfacing the placental detachment site, which was complete within 3 days of parturition (Figure 4C). Even after 4 consecutive pregnancies, we did not find evidence of appreciable gland contributions to the luminal epithelium in *Cxcl15*^*iCre*^ *R26*^*nTnG*^ mice (Figure 4D). Taken together, these data indicate that epithelial remodeling during and after pregnancy involves minimal contribution from glandular lineages and instead proceeds through expansion of luminal epithelium smiles with extensive structural similarity to menstrual-like shedding (Figure 4E).

## Discussion

Epithelial regeneration is central to endometrial function, and several models have been proposed regarding the cellular source of regenerated epithelium. Using lineage tracing with *Cxcl15*^*iCre*^ mice, we find that glandular lineages make minimal contributions to the luminal epithelium across induced menstruation, gestation, and postpartum repair in mice, challenging the prevailing model that *Axin2*-positive glandular progenitors are the primary drivers of endometrial re-epithelialization.^14,16^ This discrepancy may arise from technical limitations of lineage tracing: although *Axin2*-mediated labeling is largely gland-restricted, rare luminal cells may generate labeled patches that are not of glandular origin. An alternative progenitor is proposed to reside at the intersection zone between glands and luminal epithelium,^17^ and may be among the CXCL15-negative cells that participate in luminal resurfacing. Non-epithelial cells including fibroblasts, pericytes, and bone marrow-derived cells, have also been proposed to generate a small portion (up to 17%) of the epithelium through trans-differentiation.^31–34^ However, due to the absence of luminal epithelium-specific tracing tools, we cannot perform a definitive assessment of the relative contributions of luminal progenitors compared to potential non-epithelial sources. Nonetheless, our observations of extensive, proliferative epithelial sheets (“smiles”) abutting the lumen support a model in which the luminal epithelium serves as a major cellular source driving endometrial repair.

Our data indicate that the luminal epithelium plays a proactive role in endometrial remodeling events. During menstruation and pregnancy, we find that luminal epithelial cells proliferate, and potentially migrate^35^ to envelop decidualized tissue, and cover the underlying stroma as the smiles expand. This contrasts with the prevailing model of menstruation, which involves substantial stromal exposure followed by re-epithelialization. Equivalent expansion of luminal epithelial smiles around gestational decidua also minimizes stromal exposure by the time of parturition. Our data indicate that menstruation and gestation share common re-epithelialization mechanisms potentially mediated by signaling from the decidua. Intriguingly, *Blimp1* knockout mice fail to both encapsulate and shed the decidua during pharmacological pregnancy termination.^36^ This raises the possibility that luminal epithelial enwrapping may be functionally connected to decidual detachment, although future studies will be needed to distinguish cause and effect.

The mechanisms we identified here may not be limited to *Mus musculu*s. Indeed, observations from other mammals provide tantalizing suggestions that similar events could operate in a variety of menstruating species. Revisiting drawings from the 1950s depicting the *Elephantulus* (elephant shrew) endometrium during menstruation^37^ reveals structures and regional epithelial morphologies that are highly similar to the luminal epithelium smiles we observe. Interestingly, the only known menstruating rodent, *Acomys* (spiny mouse), has been found to lack endometrial glands altogether,^38^ raising the possibility that luminal epithelium-dominant mechanisms of endometrial re-epithelialization may play a significant role in this species.

In humans, there is evidence that glandular cells in the basalis have progenitor potential *in vitro*,^10,13^ and rebuild portions of the functionalis glands disrupted during menstruation.^15^ Consistent with a role for glands in luminal resurfacing, epithelial growths have long been observed from truncated gland stumps in human menstruation.^7–9^ Previous studies also support a role for the luminal epithelium, alongside the glands, in human menstrual re-epithelialization. Human menstrual shedding occurs in a piecemeal manner,^39^ resulting in remnant populations of luminal epithelium that are proposed to migrate to cover nearby denuded areas.^35,40^ Notably, our data from the mouse indicate that persistent luminal epithelium will preferentially resurface tissue if available, but extensive loss of luminal epithelium evokes gland contributions. Progenitor choice in the human endometrium may also be plastic, as menstruation and parturition exhibit different degrees of luminal epithelium loss. During menstruation, tissue is lost in chunks of varied size and composition, while parturition involves uniform luminal epithelium denudation.^41,42^

Although the respective contributions of glandular and luminal populations to human menstruation are challenging to determine, the potential for luminal epithelium participation has important implications for endometrial pathologies. Glandular progenitors have been favored as cells of origin for endometrial cancers and endometriosis,^43–45^ but our data indicate possible progenitor function of the luminal epithelium that could seed tumors or endometriosis lesions. The stromal resurfacing mechanisms we define here may also have relevance to heavy menstrual bleeding, which is associated with deficient endometrial repair. Our findings highlight luminal epithelial dynamics as a potential contributor to the development and progression of endometrial pathologies.

Beyond disease, our work reveals a broader regenerative principle: the mechanisms we identified provide an elegant solution for tissue elimination at massive scale, while partitioning and protecting sensitive stromal tissues. As disruption of stromal fibroblasts is often associated with scarring,^6,46–48^ minimizing the amount of tissue that needs to be re-epithelialized after decidua detachment may contribute to the endometrium’s capacity for scar-free repair. The endometrium is uniquely positioned to deploy pre-emptive epithelialization as a regenerative strategy. In most other tissues, regeneration and injury are intrinsically uncoupled, as injury arises from unpredictable external assaults. In contrast, the endometrium programs its own injury during menstruation and pregnancy, enabling it to coordinate extensive repair before tissue excision is complete.

## Methods

All key resources used in this study are listed in Table S2

### Animal husbandry

All *Mus musculus* experiments were approved by the Harvard University Institutional Animal Care and Use Committee and the University of Missouri’s Institutional Animal Care and Use Committee and adhered to all relevant ethical regulations and guidelines. All mice were housed with a 14:10 hour light-dark cycle maintained at 21 – 25°C and 30 – 70% humidity, with food (irradiated LabDiet Prolab Isopro RMH 3000 5P75; LabDiet, St. Louis, MO) and water available *ad libitum.* Female mice were group-housed with no more than 5 per cage (7.75” W × 12” L × 6.5” H), with Coarse Grade Sani-Chip Bedding (P.J. Murphy, Ladysmith, WI) and used for experiments between 8 and 21 w of age. When indicated, induction of pseudopregnancy (a physiological state of high progesterone) was achieved by pairing females with single vasectomized CD-1 males. The day the copulatory plug was found was designated pseudopregnancy day 0.5. For induction of pregnancy, females were paired with single C57BL/6J fertile males and checked for the presence of a copulatory plug every morning and evening until found. The day the copulatory plug was found was designated gestational day 0.5. For fertility testing and postpartum experiments, pregnant dams were checked each morning for new litters starting on gestational day 17.5. Pups were counted when relevant. For postpartum experiments, the day the litter was found was considered postpartum day 0.5. All experiments were performed with at least n = 3 mice per condition.

### Tissue collection

To label proliferating cells *in vivo*, mice received an intraperitoneal injection of 25 μg/g EdU 1 h prior to tissue harvest. Animals were either euthanized by 5 min exposure to CO_2_ or by cervical dislocation. Uterine horns were promptly excised with ovaries, cervix and mesometrium attached. Horns were laid flat on filter paper, briefly imaged in a 10” × 10” LED lightbox to document gross morphology, then fixed in ice cold 4% paraformaldehyde overnight at 4°C with gentle agitation. Samples were then washed 3 times, 15 min – 2 h each in PBS, and trimmed of any excess fat or mesometrium as needed, before storage at 4°C in 0.1% azide in PBS until use.

### Lineage tracing experiments

Uteri from *Cxcl15*^*iCre*^ *R26*^*nTnG*^ females were collected at the following time points for lineage tracing experiments:

- Homeostasis: afternoon of pseudopregnancy day 4.5.
- Chemical ablation: polidocanol injection was performed when animals were in estrus, and horns were collected for further analysis 2 h, 72 h, 14 d, or 28 d later.
- Gestation: gestational day 7.5 or 8.5 between 12:00pm and 4:00pm.
- Postpartum: postpartum day 0.5 or 3.5 between 12:00pm and 4:00pm.

### Luminal epithelium chemical ablation

C57BL/6 or *Cxcl15*^*iCre*^ *R26*^*nTnG*^ females in the estrus stage of the hormonal cycle were identified by the ratio of leukocytes, nucleated epithelial cells, and cornified epithelial cells present in vaginal lavage fluid. Polidocanol foam was generated by combining 4 parts air and 1 part 5% polidocanol in syringes attached with a luer lock adapter and passing the mixture through the adapter 60 times each way. Polidocanol foam or saline (for negative controls) was injected into the uterine lumen from the cervical third of the horn using a 32G needle until visual confirmation that it filled the entire horn, then the process was repeated for the contralateral horn. Any foam that leaked out of the injection site was thoroughly flushed away with sterile saline. The foam or saline was allowed to sit in the uterine lumen for 15 min to facilitate full ablation of the luminal epithelium. During this time, the abdominal cavity was covered with sterile gauze to limit exposure to the environment, and routinely irrigated with warmed sterile saline to keep the tissue hydrated. After 15 min, the foam was flushed out of the lumen by injecting 250 μL sterile saline per horn using a 32G needle to limit further ablation. Residual polidocanol foam was thoroughly flushed from the outside of the uterus, abdominal fat pads and skin around the incision site with sterile saline immediately after polidocanol injection and intrauterine saline flushing. The uterus was then returned to the abdominal cavity and the incision was closed. Uterine horns were collected at the indicated time points or animals were allowed to recover for 4 days for fertility testing (see Figure 2E for experimental timeline). All mice used for this assessment exhibited copulatory plugs within 9 days of polidocanol treatment.

### Oil induction of menstruation in ovariectomized mice

*Cxcl15*^*iCre*^ *R26*^*nTnG*^ females were bilaterally ovariectomized, then allowed to recover for 7 – 14 d (see Figure S1A for an experimental timeline). Beginning on day 0, mice received 3 consecutive days of subcutaneous estradiol (100 ng in sesame oil), then were allowed to rest for 2 days. Mice then received daily subcutaneous injections of 6.7 ng of estradiol and 1 mg progesterone in sesame oil from days 5 – 11. To induce decidualization, mice received an intrauterine oil injection on the evening of day 7. Each horn received 50 μL of sesame oil or sterile saline (for negative controls) using a 31G insulin syringe inserted into the third of the horn proximal to the cervix. To assess bleeding, daily vaginal lavages were collected in 30 – 100 μL sterile saline from mice beginning the day after intrauterine oil injection. Lavage fluid was tested for the presence of red blood cells using guaiac feccal occult blood tests. Animals were harvested 10 d after the last estradiol and progesterone injection, or on the day of their first robust positive guaiac test. Animals that exhibited positive guaiac tests before the last progesterone injection were omitted from the study.

### Chemogenetic induction of menstruation in X-Mens mice

We used the previously described method of chemogenetic induction of decidualization and menstruation in X-Mens mice.^28^ Pseudopregnancy was induced in non-ovariectomized *Amhr2*^*Cre*^ *R26*^*GsD*^ females to raise progesterone levels (see Figure S1C for an experimental timeline). On the evening of pseudopregnancy day 3.5, animals received drinking water with 48 μg/mL clozapine-N-oxide dihydrochloride (CNO) after 4:00 pm. On day 4.5, animals were given 5 consecutive intraperitoneal injections of a DREADD agonist cocktail of 24 μg/mL CNO, 125 μg/mL deschloroclozapine dihydrochloride, and 200 μg/mL compound 21 dihydrochloride in physiological saline, spaced 2h apart. The CNO drinking water was replaced with regular drinking water by 11:00 am the following day. In this model, DREADD activation under a high progesterone state induces decidualization. As the pseudopregnancy resolves, progesterone levels will naturally decline, which triggers decidual shedding. To assess shedding and bleeding, daily vaginal lavages were collected in 30 – 100 μL sterile saline from mice beginning the day after DREADD agonist cocktail administration. Lavage fluid was tested for the presence of red blood cells using guaiac feccal occult blood tests, and testing continued until blood was no longer detected in the lavage fluid for 2 consecutive days, or until the uterus was collected. Animals harvested at the first day of bleeding were harvested on the day of their first robust positive guaiac test.

### Consecutive pregnancies

*Cxcl15*^*iCre*^ *R26*^*nTnG*^ females (6 – 8 weeks of age) were housed individually and continuously with a fertile *Cxcl15*^*iCre*^ *R26*^*nTnG*^ male to allow uninterrupted breeding. Females were maintained with the male until four litters were produced, after which the male was removed. The fourth litter was weaned at postnatal day 21, and uteri were collected from the dam during the subsequent diestrus. The tissue was harvested and fixed in 4% paraformaldehyde in PBS for 60 min at 4 °C, followed by cryoprotection by overnight immersion in 15% sucrose in PBS at 4 °C. Tissue was embedded in optimal cutting temperature compound (OCT) and cryosectioned at 7 – 10 μm. Sections were mounted on Superfrost Plus slides, counterstained with 2 μg/mL Hoechst 33342, and coverslipped with ProLong^™^ Diamond Antifade Mountant. Images were acquired using a Leica DM6 B upright microscope equipped with a Leica K8 camera and Leica Application Suite X (LAS X) software.

### Tissue cryosectioning and immunostaining

Following overnight fixation, individual uterine horns were transferred to 30% sucrose in PBS and incubated overnight at 4 °C for cryoprotection. Tissues were then embedded in OCT compound, snap frozen, and stored at −80 °C until use. OCT blocks were equilibrated in the cryostat chamber at −19 to −21 °C for a minimum of 30 min prior to sectioning. Uterine horns were sagittally sectioned at 12 μm on a Leica CM 1860 cryostat, proceeding from the cervical toward the ovarian end. Sections were collected onto positively charged glass slides and stored at −80 °C.

For immunofluorescence, slides were post-fixed in 4% formaldehyde in PBS for 5 minutes at room temperature, then washed three times in PBS with 0.1% Tween-20 (PBT), 15 min per wash. Sections were incubated for 30 – 60 min at room temperature in blocking buffer (PBS containing 5% normal donkey serum, 0.5% BSA, 0.1% Triton X-100, and 0.1% sodium deoxycholate). Section perimeters were outlined with a hydrophobic barrier pen. Sections were incubated with primary antibodies diluted in blocking buffer overnight at 4 °C in a humidified chamber, then washed three times in PBT. Sections were then incubated in blocking buffer containing secondary antibodies and DAPI (1 μg/mL) overnight at 4 °C in a humidified chamber. Slides were washed three times in PBT, then coverslipped with ProLong Gold Antifade mounting medium and allowed to cure for at least 24 h at room temperature. All antibodies used are listed in Table S2.

### Thick section tissue clearing, EdU staining, and immunostaining

Single uterine horns were mounted in 2–5% low gelling temperature agarose, then sectioned with a vibratome or compresstome at 200 μm thickness and stored in PBS with 0.1% azide at 4° C until use. All steps were carried out at room temperature, unless noted otherwise. Sections were first incubated at 37°C in permeabilization solution 1 (0.2% TritonX-100, 10% DMSO, 0.2% Deoxycholate, 20 mM EDTA in PBS, pH 8.0) for 24 h, then transferred to permeabilization solution 2 (0.2% Triton X-100, 10% DMSO, 100 mM glycine in PBS) for 1 h at 37°C. X-Mens samples containing endogenous fluorescent proteins required an additional bleaching step, in which sections were incubated in 10% hydrogen peroxide in 0.05 M phosphate buffer at 37°C for 3 h, then quickly washed twice in PTwH (0.2% Tween-20, 10 μg/mL heparin in PBS), followed by three longer washes in PTwH, 10 min each. For EdU staining, sections were quickly washed twice in 3% BSA in PBS, then incubated for 1 h in a freshly made reaction cocktail (2 mM Copper sulfate pentahydrate, 8 μM sulfo-cyanine3 azide and freshly dissolved 113 mM ascorbic acid in PBS, pH 7.4) and then quickly washed twice with 3% BSA before proceeding to blocking. Sections were blocked in 0.2% Triton X-100, 10% DMSO, 5% Normal Donkey Serum in PBS for 1 h, then incubated with primary antibodies diluted in a staining solution of 0.2% Tween-20, 10 μg/mL heparin, 5% DMSO, and 5% Normal Donkey Serum in PBS at 4°C overnight. Sections were then quickly washed twice with PTwH, then three times with PTwH, 10 min each, then allowed to incubate for 3 h with the appropriate donkey secondary antibodies. Staining solutions contained DAPI diluted 1:1000 to label nuclei. Sections were then washed in PTwH twice for a few seconds each, followed by three washes of 10 min each. Next, sections were optically cleared to help facilitate imaging through the full thickness as follows: after removing as much liquid as possible, sections were incubated in an EZ index solution of 80% Nycodenz, 42% Urea and 0.025% sodium azide in 0.2 M, pH 7.4 phosphate buffer for at least 1 h. Sections were mounted in EZ index on slides fit with 200 μm thick spacers, and imaged within 4 d of mounting. All antibodies used are listed in Table S2.

### Imaging and image processing

To show multiple gland-luminal epithelium junctions, uterine immunofluorescence images are shown as maxIPs of 200 μm-thick transverse sections, unless noted otherwise in the figure legend. Immunostained uterine sections were imaged on a Yokogawa CSU-W1 spinning disc confocal system fit to an inverted Nikon Ti-2E microscope. All images were acquired using a CFI60 Plan Apochromat Lambda 20x/0.75 NA objective. Thick sections were imaged with a 5 μm Z-step size, and cryosections were imaged with a 0.9 μm Z-step size. Large images were acquired using Nikon Elements JOBS module. Stitched images and maximum intensity projections were generated using Nikon Elements General Analysis 3 module. PNGs were generated using Fiji/ImageJ.

### Quantification and statistical analysis

For all experiments, at least three independent replicates were examined. For each replicate, three 200 μm-thick sections collected from the cervix-proximal, middle, and ovary-proximal thirds of each uterine horn were examined. Data are expressed as mean +/− standard deviation. All images were analyzed in Fiji/ImageJ.^54^

For quantification of the proportion of GFP-positive glandular epithelium in *Cxcl15*^*iCre*^ *R26*^*nTnG*^ samples, 200 μm-thick sections were immunostained for FOXA2 and GFP. For each section, three equally spaced Z-planes were selected for analysis. FOXA2-positive cells were first manually annotated using the multi-point tool. After annotating all FOXA2-positive cells on a given section, the GFP channel was toggled on and the previously marked FOXA2-positive cells were re-inspected; cells were scored as double-positive (FOXA2^+^GFP^+^) when GFP signal overlapped with the same cell. Any candidate cell with a mean pixel intensity less than 300 (arbitrary units) was deemed too faint to accurately classify and excluded from analysis.

For quantification of the proportion of GFP-positive luminal epithelium in *Cxcl15*^*iCre*^ *R26*^*nTnG*^ samples, three 12 μm-thick cryosections per uterine horn (spaced at least 300 μm apart) were immunostained for GFP and imaged. GFP immunostaining was imaged in the 640 channel to maximize the ratio of signal to noise. The mean length of individual luminal epithelial nuclei was determined to be 0.475 μm by quantifying the number of cells spanning a 40 μm luminal segment across randomly selected regions; 21 regions in total were measured across 7 sections (3 regions per section). The perimeter of the uterine lumen was then traced in each section using the polygon selection tool in Fiji, and the total luminal epithelial cell number was estimated by dividing the measured perimeter by the mean cell length. GFP-positive cells were counted manually. All quantification data are provided in Table S1.

For smile frequency quantification, entire uterine horns were serially sectioned at a 200 μm-thickness. Every other section was immunostained for EPCAM and examined for the presence of continuous EPCAM-positive cells (sheets) around at least a portion of the periphery of the decidua. Damaged sections were excluded from analysis.

## Supplementary Material

Supplement 1

## Data Availability

Requests for further information and resources should be directed to and will be fulfilled by the lead contact, Kara McKinley (kara_mckinley@harvard.edu). This study did not generate new unique reagents or code.
