# Supplementary material for: Luminal epithelium remodeling underlies endometrial regeneration during menstruation and pregnancy
Source: bioRxiv. 2026 Mar 10:2026.03.08.710375. Preprint. [Version 1] doi: 10.64898/2026.03.08.710375 (PMC13034174; doi:10.64898/2026.03.08.710375)
Supplement: Supplement 1 [file media-1.pdf]

Luminal epithelium remodeling underlies endometrial regeneration during menstruation and pregnancy

This file includes
Figures S1 and S2
Tables S1 and S2

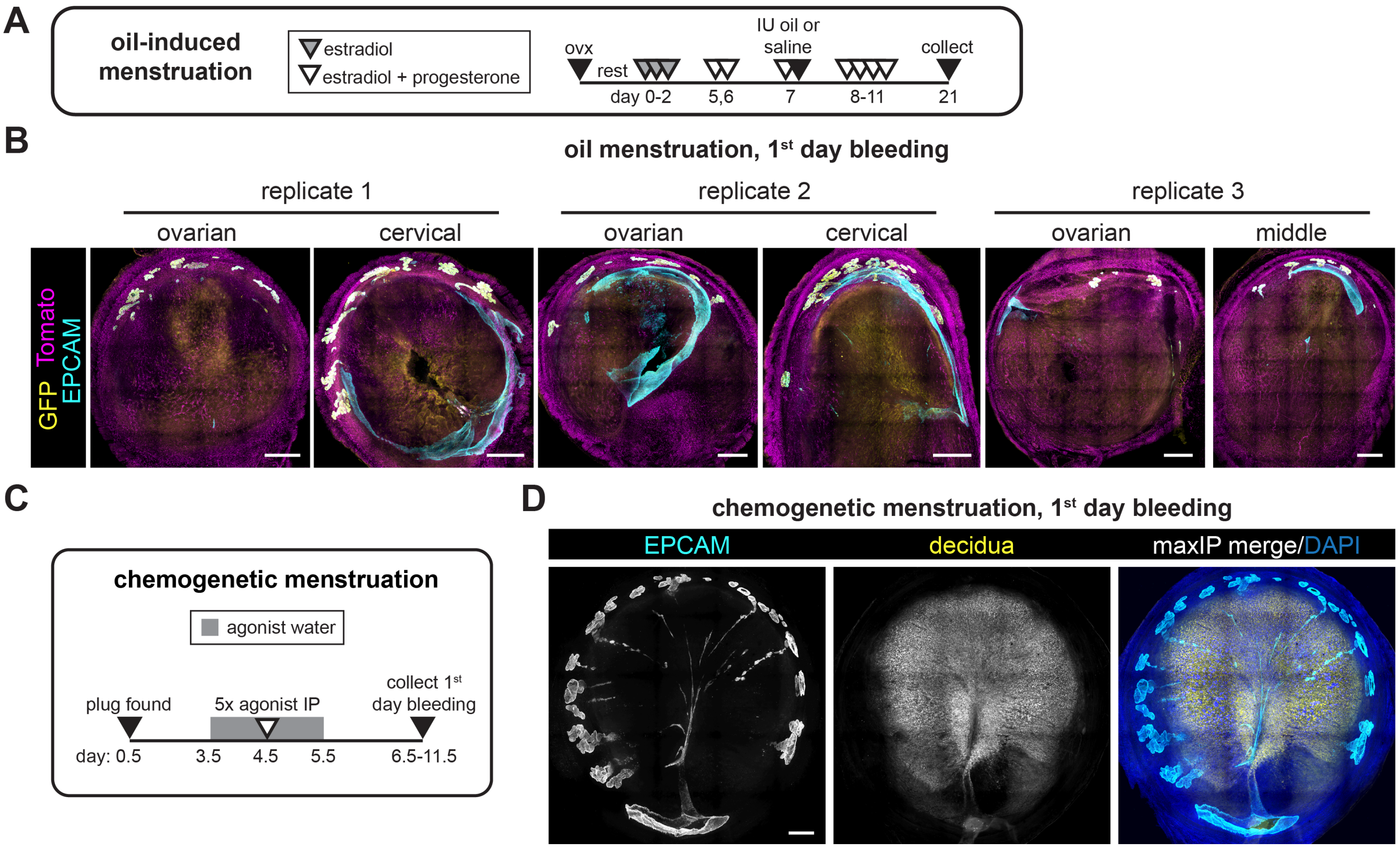

Figure S1. Morphogenic changes to luminal epithelium throughout menstruation, related to Figure 3.
(A) Experimental timeline for oil induction of menstruation in ovariectomized *Cxcl15*^{iCre} *R26*^{nTnG} mice.
(B) Additional examples of immunofluorescence images of *Cxcl15*^{iCre} *R26*^{nTnG} uterine sections collected on the first day of oil-induced menstrual bleeding. Sections were examined from three regions of each uterine horn: ovary-proximal, middle, and cervix-proximal. The middle section from replicate 2 is shown in Figure 3C. LUTs were adjusted independently to maximize signal-to-noise. Scale bars, 500 μm.
(C) Experimental timeline for chemogenetic induction of menstruation in X-Mens mice.
(D) Representative immunofluorescence images of an X-Mens uterus exhibiting a smile structure, collected as indicated in C. 27% of samples exhibited smiles, and all sections examined contained persistent luminal epithelium.
IP, intraperitoneal.

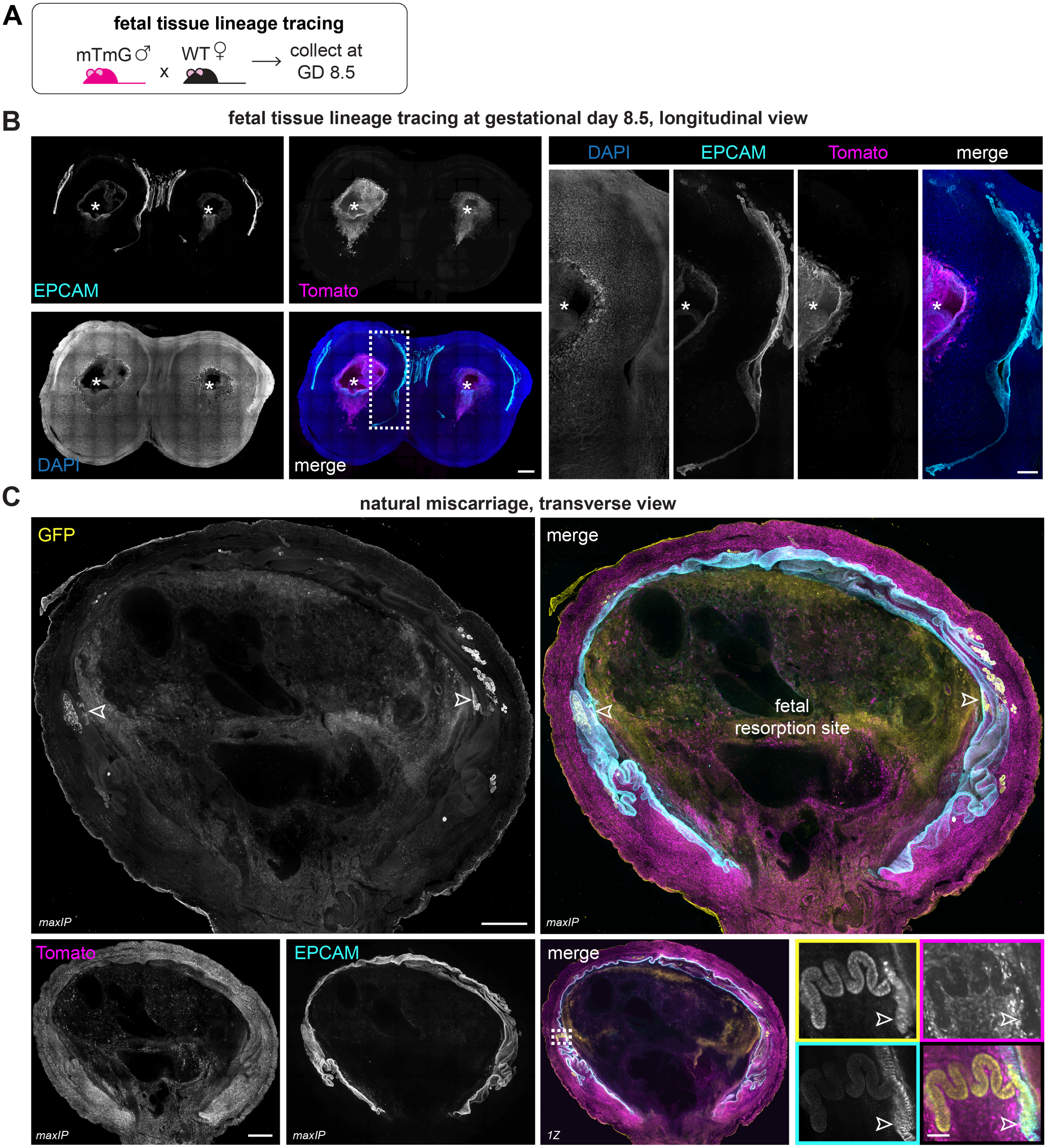

Figure S2. Luminal epithelium contributions to gestational remodeling and fetal resorption, related to Figure 4.
(A) Experimental paradigm for lineage tracing fetal-derived tissue.
(B) Representative immunofluorescence maxIP images of two adjacent longitudinally sectioned implantation sites, collected as indicated in A. Outset scale bars, 500 μm; inset scale bars, 200 μm.
(C) Single immunofluorescence image of a naturally arising fetal-resorption site collected on the day of parturition. Outlined arrowhead indicates rare GFP-positive luminal epithelial cells. Outset scale bars, 500 μm; inset scale bars, 50 μm.
Insets are shown as single Z planes. Inset channel is indicated by box color unless directly noted. Outlined arrowheads indicate GFP-positive luminal epithelial cells, and asterisks indicate embryos. Outset scale bars, 500 μm; inset scale bars, 50 μm.
GD, gestational day; WT, wildtype.

Mouse ID	Avg number of GFP+ luminal epithelium nuclei per section	Avg number of luminal epithelium nuclei per section	Percent GFP+ luminal epithelium nuclei per section	Avg percent GFP+ luminal epithelium nuclei across condition ± std dev
**Homeostasis, 9w of age**				
L524	0.67	6,052.14	0.01	0.09 ± 0.09
L525	12.3	6,484.77	0.19	
L527	2.67	4,554.60	0.06	
**Homeostasis, 21w of age**				
L522	7.00	3,681.19	0.20	0.19 ± 0.01
L526	6.00	3,585.47	0.17	
L874	9.50	4,271.05	0.19	
**Oil-induced menstruation, control**				
U317	8.50	8,164.38	0.10	0.06 ± 0.05
U492	4.00	4,360.32	0.09	
Q123	0.00	2,512.91	0.00	
Q124	1.33	3,143.17	0.05	
**Oil-induced menstruation, menstruated**				
Q643	4.33	3,328.54	0.13	0.27 ± 0.14
Q644	3.00	2,451.30	0.13	
U315	21.33	7,592.21	0.31	
U817	7.67	3,439.26	0.26	
U818	15.33	2,849.64	0.55	
U819	8.00	1,856.68	0.32	
U820	5.00	1,744.84	0.28	
V276	11.33	7,644.70	0.15	

Table S1. Percent GFP-positive luminal epithelium across homeostasis and menstruation, related to Figures 1 and 3.
Quantification of the proportion of luminal epithelial nuclei expressing GFP in homeostatic conditions, and after recovery from oil-induced menstruation. Values for each mouse are averaged across three cryosections.

Table S2. Materials used in this study		
REAGENT or RESOURCE	SOURCE	IDENTIFIER
**Antibodies**		
Chicken polyclonal anti-GFP; 1:1000	Abcam	Cat# ab13970; RRID: AB_300798
Goat polyclonal anti-P-cadherin; 1:200	R&D Systems	Cat# AF761; RRID: AB_355581
Goat polyclonal anti-TdTomato; 1:1000	SICGEN	Cat# AB8181; RRID: AB_2722750
Rabbit monoclonal anti-FoxA2/HNF3 (clone D56D6); 1:200	Cell Signaling Technology	Cat# 8186; RRID: AB_10891055
Rat monoclonal anti-CD326/EpCAM (clone G8.8); 1:1000	BioLegend	Cat# 118202; RRID: AB_1089027
Rat monoclonal anti-Cytokeratin 8 (clone TROMA-1); 0.42 µg/mL final concentration	Developmental Studies Hybridoma Bank (Brûlet et al.[49])	Cat# TROMA-1
Donkey anti-Chicken AF488; 1:1000	Thermo Fisher Scientific	Cat# A78948; RRID: AB_2921070
Donkey anti-Goat AF488; 1:1000	Thermo Fisher Scientific	Cat# A11055; RRID: AB_2534102
Donkey anti-Goat AF555; 1:1000	Thermo Fisher Scientific	Cat# A32816; RRID: AB_2762839
Donkey anti-Goat AF647; 1:1000	Thermo Fisher Scientific	Cat# A21447; RRID: AB_2535864
Donkey anti-Rabbit AF555; 1:1000	Thermo Fisher Scientific	Cat# A31572; RRID: AB_162543
Donkey anti-Rabbit AF647; 1:1000	Thermo Fisher Scientific	Cat# A31573; RRID: AB_2536183
Donkey anti-Rat AF488; 1:1000	Thermo Fisher Scientific	Cat# A21208; RRID: AB_2535794
Donkey anti-Rat AF647; 1:1000	Thermo Fisher Scientific	Cat# A78947; RRID: AB_2910635
**Chemicals, peptides and recombinant proteins**		
Paraformaldehyde, 32%	Electron Microscopy Sciences	Cat# 15714-S
Agarose, low gelling temperature	Sigma-Aldrich	Cat# A9414
Ascorbic acid	Sigma-Aldrich	Cat# A4544
BSA (bovine serum albumin)	Sigma-Aldrich	Cat# 10775835001
CNO (clozapine-N-oxide dihydrochloride)	MedChemExpress	Cat# HY-17366A
Compound 21 dihydrochloride	MedChemExpress	Cat# HY-100234A
$CuSO_4$ $5H_2O$ (copper sulfate pentahydrate)	Sigma-Aldrich	Cat# 12849

DAPI	Thermo Fisher Scientific	Cat# 62248
DCZ (deschloroclozapine dihydrochloride)	MedChemExpress	Cat# HY-42110A
DMSO (dimethyl sulfoxide)	Tokyo Chemical Industry	Cat# D5293
EDTA (0.5 M, pH 8.0)	Thermo Fisher Scientific	Cat# 15575020
EdU (5-Ethynyl-2’-deoxyuridine)	TargetMol	Cat# T17341
17β-Estradiol	Sigma-Aldrich	Cat# E8875
Ethanol, 200 proof	Decon Labs	Cat# V1016TP
Ethiqa XR (extended-release buprenorphine)	Fidelis Animal Health	Cat# NDC 86084-100-30
Glycine	Sigma-Aldrich	Cat# G8898
Heparin	Sigma-Aldrich	Cat# H3393
Hoechst 33342	Invitrogen	Cat# H3570
Isoflurane	Akorn Inc	Cat# 07-894-6668
Low gelling temperature agarose	Sigma-Aldrich	Cat# A9414
Mifepristone	MedChemExpress	Cat# HY-13683
Normal Donkey Serum	Neuromics	Cat# SER004
Nycodenz	Serumwerk	Cat# 18003
PBS with 0.1% azide	Santa Cruz	Cat# sc-296028
Peanut oil	Sigma-Aldrich	Cat# P2144
Phosphate Buffer (0.2 M, pH 7.4)	Moltox	Cat# 26-510.047
Polidocanol (nonaethylene glycol monododecyl ether)	Sigma-Aldrich	Cat# P9641
Progesterone	Sigma-Aldrich	Cat# P8783
Sesame oil	Sigma-Aldrich	Cat# S3547
Sodium Deoxycholate	Sigma-Aldrich	Cat# 30970
Sulfo-cyanine3 azide	Lumiprobe	Cat# 1330
TritonX-100	Sigma-Aldrich	Cat# X100
Tween-20	Sigma-Aldrich	Cat# P9416
Urea	Sigma-Aldrich	Cat# U5128
**Critical commercial assays**		
Hemoccult Guaiac Fecal Occult Blood Test Systems	Beckman Coulter	Cat# 60151A
**Experimental models: Organisms/strains**		
Mouse: Amhr2^{Cre}: B6.129(Cg)-Gt(ROSA)26Sortm4(ACTB-t dTomato,-EGFP)Luo/J	Ron Chandler (Jamin et al.[50])	N/A
Mouse: B6: C57BL/6	The Jackson Laboratory	RRID: IMSR_JAX:000664
Mouse: CD-1, vasectomized	Charles River	RRID: IMSR_CRL:022

Mouse: Cxcl15^{Cre}	Andrew Kelleher (Kelleher et al.[20])	MGI ID TBD
Mouse: GsD: Gt(ROSA)26Sortm1(-CAG-Chrm3*/GFP,c AMPRE-luc)Berd	Bruce Morgan (Akhmedov et al.[51])	N/A
Mouse: mTmG: B6.129(Cg)-Gt(RO-SA)26Sortm4(ACTB-tdTomato,-EGFP) Luo/J	The Jackson Laboratory (Muzumdar et al.[52])	RRID: IMSR_JAX:007676
Mouse: nTnG: B6N.129S6-Gt(RO-SA)26Sortm1(CAG-tdTomato*,-EGFP*) Ees/J	The Jackson Laboratory (Prigge et al.[53])	RRID: IMSR_JAX:023537
**Software and algorithms**		
Fiji/ImageJ version 1.54p	ImageJ	https://imagej.net/software/fiji/
Leica Application Suite X (LAS X) software	Leica	https://www.leica-microsystems.com/products/microscope-software/p/leica-las-x-ls/
NIS-Elements version 5.42.06	Nikon Instruments	https://www.microscope.healthcare.nikon.com/products/software/nis-elements
**Other**		
CFI60 Plan Apochromat Lambda 20×/0.75 NA objective lens	Nikon Instruments	Cat# MRD00205
Compresstome® (vibrating microtome)	Precisionary Instruments	Cat# VF-510-0Z
Cryostat CM 1860	Leica	N/A
CSU-W1 spinning disk confocal system	Yokogawa	Cat# 99927
DM6 B upright microscope	Leica	https://www.leica-microsystems.com/products/light-microscopes/p/dm4-b-and-dm6-b-upright-microscopes/
Hydrophobic barrier pen	Vector Laboratories	Cat# H-4000
Inverted Ti-2E microscope	Nikon Instruments	Cat# MEA54000
K8 camera	Leica	https://www.leica-microsystems.com/products/microscope-cameras/p/k8/
ORCA-Fusion Gen-III sCMOS camera	Hamamatsu	Cat# 77054115
ProLong™ Diamond Antifade Mountant	Invitrogen	Cat# 36961
ProLong™ Gold Antifade Mountant	Thermo Fisher Scientific	Cat# P36930
Somnosuite (low-flow anesthesia system)	Kent Scientific	Cat# SS-01

Superfrost Plus slides	Thermo Fisher Scientific	Cat# 22037246
Tissue-Tek® OCT compound	Sakura	Cat# 4583
VT1000 S Vibratome	Leica	RRID: SCR_016495

Table S2. Key resources used in this study.
